# Differences and Similarities between Perpetrators of Ethnic and Non-Ethnicity-Based Victimization

**DOI:** 10.1007/s10964-020-01271-5

**Published:** 2020-06-25

**Authors:** Sevgi Bayram Özdemir, Clover Giles, Metin Özdemir

**Affiliations:** 1 Center for Lifespan Developmental Research, School of Law, Psychology and Social Work, Örebro University, 701 82 Örebro, Sweden; 2 School of Law, Psychology and Social Work, Örebro University, 701 82 Örebro, Sweden

**Keywords:** Ethnic victimization, Moral disengagement, Racial bullying, Ethnic bullying, Empathy, Impulsivity, Anti-immigrant attitudes

## Abstract

Immigrant and minority youth are at risk of ethnic victimization. Despite an increasing number of studies that aim to understand the consequences of such negativity, relatively little attention has been paid to understanding who the perpetrators of ethnic victimization are. To address this gap in knowledge, the present study examined whether youth who victimize their peers due to their ethnic background are also those who engage in non-ethnicity-based victimization. The study also investigated the underlying factors, i.e., impulsivity, empathy, moral disengagement, and attitudes toward immigrants, that are common or specific to groups of youth. The sample included 949 adolescents residing in Sweden (*M*_age_ = 13.11, SD = 0.41; range: 12–15; 46% girls). Cluster analysis revealed four distinct groups of adolescents, based on their reports of ethnic and non-ethnicity-based victimization: (1) low on both forms of victimization, (2) high on ethnic victimization only, (3) high on non-ethnicity-based victimization only, and (4) high on both forms of victimization. The results showed that being morally disengaged is a common denominator of ethnic and non-ethnicity-based victimizers. Difficulties in regulating impulses and lack of perspective-taking skills trigger youth’s engagement in non-ethnicity-based victimization. Lack of empathic concerns and low levels of positive attitudes toward immigrants are the bases of ethnic victimization. Together, these findings suggest that the precursors of ethnic and non-ethnicity-based victimization have similarities as well as differences, which require further attention in developing programs aimed at preventing different forms of peer victimization.

## Introduction

Promoting harmonious inter-ethnic relationships is a key challenge for immigrant-receiving countries. Unfortunately, a number of studies have shown that immigrant youth are at risk of ethnicity-based victimization, which prompts them to regard themselves as not welcomed or valued in the host society. For example, immigrant youth in Sweden, particularly first-generation non-Europeans, report more social rejection and isolation than their majority peers (Plenty and Jonsson 2017). Similarly, in the Netherlands, it was found that 42% of ethnic-minority children aged 10–13 had experienced racist name calling in school, twice the number of their majority peers. Moreover, up to 30% of minority children were subjected to ethnicity-motivated social exclusion (Verkuyten and Thijs 2002). These negative experiences have detrimental consequences for immigrant and minority youth’s psychosocial and behavioral adjustment (Bayram Özdemir et al. 2019; Priest et al. 2013), and may jeopardize their integration into the host society (Marks et al. 2015). Despite a growing body of research on ethnic victimization, it is still unclear who the perpetrators of ethnic victimization are (for exceptions see, e.g., Bayram Özdemir et al. 2018; Caravita et al. 2019). Even more importantly, it is unknown whether the youth who victimize their peers due to their ethnic background are also the ones who engage in non-ethnicity-based victimization. Knowledge is needed to understand how similar or different the perpetrators of ethnicity and non-ethnicity-based victimization are, and to identify the common and differential causes of their behaviors. Such knowledge may eventually lead to taking of effective measures to prevent different forms of victimization, and in turn, to increase social cohesion in schools.

To address this important gap in knowledge, the first goal of this study was to investigate whether there are distinct groups of adolescents who engage in ethnic and/or non-ethnicity-based victimization. Its second goal was to elucidate the underlying factors that are common or unique to each group. These overarching study goals were investigated within the Swedish cultural context. Sweden is among the countries that emphasize freedom of thought and equality of opportunities (World Value Survey 2015), and has been ranked as the country with the strongest integration-promoting polices in the world (MIPEX 2015). In addition, low numbers of school-bullying incidents have been reported in Sweden compared with other European and North America countries (Craig et al. 2009). For example, recent PISA results showed that 19% of 15-year-old students reported being bullied at least a few times a month in Sweden, compared with 23% on average across OECD countries (PISA 2018). Despite its promising profile on migration policies in large-scale international surveys, and its lower prevalence of self-reported bullying, Swedish society has also experienced increased polarization due to immigration crisis and growing anti-immigrant ideologies in the European Union. Relatedly, recent large-scale surveys indicate that students of foreign background are at greater risk of negative peer treatment (BRÅ 2017; Bjereld et al. 2015), particularly in relation to their background. Accordingly, systematic action, informed by scientific evidence, is urgently needed to counteract the problem.

### Why Do Adolescents Engage in Ethnic and/or Non-Ethnicity-Based Victimization?

Adolescence is a critical period when the bases of global competencies are formed. In particular, young people explore their self and identity during this developmental period (McLean and Syed 2015), and also develop ideas about others. On average, they become more cognitively mature, which may help them adopt more abstract reasoning, and develop perspective-taking skills and prosocial moral reasoning (Eisenberg et al. 2005). Further, peer acceptance becomes salient to adolescents, and, in turn they demonstrate a greater sensitivity to peer relationships and peer groups (Brown and Larson 2009). Thus, understanding why young people engage in ethnic and/or non-ethnicity-based victimization during this period would be informative in identifying ways to intervene with the problem early on, and in promoting social interactions among diverse groups of people later in life. In light of major theoretical and conceptual arguments in the aggression-and-bullying (Gottfredson and Hirschi 1990; van Noorden et al. 2017; Hymel et al. 2005) and ethnic-victimization literature (Bayram Özdemir et al. 2016; Caravita et al. 2019), the present study examined the extent to which youth’s ability to regulate their behaviors (i.e., control their impulsivity) and to feel and understand others’ feelings (i.e., their empathy), their moral justification for their behaviors, and their attitudes toward immigrants explained their engagement in ethnic and/or non-ethnicity-based victimization.

#### The role of impulsivity

Self-control theory posits that certain dispositions restrict people from regulating their impulses and refraining from problem behaviors (Gottfredson and Hirschi 1990). Specifically, the theory suggests that people with poor self-control, including those with impulsive personality traits, are lacking in the ability to thoroughly consider the pros and cons of their behaviors, for themselves and others. Impulsive individuals are less likely to delay their own gratification, and are therefore at greater risk of engaging in problem behaviors. Supporting the premises of self-control theory, empirical studies have shown that impulsive adolescents have difficulties in regulating their anger and overly react to minor frustrations (Colder and Stice 1998). These adolescents have also been found to display aggressive behaviors (Fanti and Kimonis 2012), engage in delinquent (see Vazsonyi et al. 2017 for a meta-analysis) and violent behaviors (Bayram Özdemir et al. 2016, 2019), have positive attitudes toward bullying (Walters et al. 2018), and be more likely to harass (Bayram Özdemir et al. 2016) or bully their peers in school (Chui and Chan 2013; Jolliffe and Farrington 2011). All this applies especially with increasing age (see Geel et al. 2017 for a meta-analysis). Together, these findings suggest that impulsivity is a critical predisposition that puts young people at risk of engaging in various forms of problematic externalizing behavior, including victimizing their peers in school.

Despite a well-established literature on the link between impulsivity and youth’s engagement in antisocial behaviors, the extent to which impulsivity plays a role in youth’s engagement in aggression in inter-ethnic relationships, including ethnic victimization, has not been thoroughly examined. There are only two empirical studies in this regard, which have yielded somewhat similar findings. Specifically, the first study focused on Swedish adolescents and showed that youth with higher levels of impulsivity were more likely to engage in ethnic harassment (conceptualized as harassing peers due to their ethnic and/or cultural background), especially if they also held anti-immigrant views (Bayram Özdemir et al. 2016). However, it should be noted that the magnitude of the association between impulsivity and engagement in ethnic harassment was small (*r* = 0.10), and it became non-significant after controlling for other factors, such as adolescents’ demographic characteristics and attitudes toward immigrants. The second study examined the extent to which a lack of self-control (in which impulsivity is one aspect of the overall construct) contributes to engagement in hate-motivated, as opposed to non-hate-motivated, assaults and bullying among ninth-grade students in Finland. It was shown that adolescents with a lack of self-control had a greater likelihood of engaging in both hate-motivated and non-hate-motivated bullying, even after controlling for adolescent gender, family socio-economic status, and parental supervision. Interestingly, the magnitude of the effect was twice as high for non-hate-motivated than hate-motivated bullying (Näsi et al. 2016). Together, these findings suggest that impulsivity may contribute to engagement in general victimization to a greater extent than it does to ethnic victimization.

#### The role of empathy

Empathy is a complex multidimensional phenomenon, which comprises the automatic and directed emotional and cognitive responses that allow an individual to experience and comprehend the inner life of others (Davis 1983). The cognitive component of empathy (referred to as perspective-taking) is characterized as the ability to *understand* another person’s perspectives and feelings, whereas the emotional component of empathy (referred to as empathic concern) reflects the ability to *feel or experience* what another person is feeling. It has been posited that people who lack empathy may act without thoroughly understanding and experiencing how their behaviors might impact others’ emotions. Accordingly, those who lack empathy may be more prone to having problems in their relationships, including interpersonal aggression and engagement in bullying. Supporting this view, some studies have shown that youth who lack *affective* empathy are more likely to bully their peers in school (see van Noorden et al. 2017 for a meta-analytical review; Zych and Llorent 2019), and to assist and reinforce the behaviors of bullies (e.g., Gini et al. 2015; see Mitsopoulou and Giovazolias 2015 for a review), and are less likely to defend the victims of bullying (see van Noorden et al. 2017 for a meta-analytical review; Longobardi et al. 2019). However, findings on the association between *cognitive* empathy and antisocial behavior are somewhat inconclusive. Whereas some studies have indicated a negative association between cognitive empathy and aggressive behavior (e.g., Dinić et al. 2016), others have shown no association (e.g., Jolliffe and Farrington 2011; Stavrinides et al. 2010). Together, the findings suggest that children who victimize their peers are not necessarily incapable of comprehending what their peers feel, but rather that they have an impaired ability to experience what their peers might feel, which indicates that lack of affective empathy may be an important precursor of engagement in peer victimization.

Despite an extensive body of literature on the role of empathy in adolescents’ engagement in antisocial behaviors, how (lack of) empathy contributes to adolescents’ interactions in ethnically diverse settings has not been extensively examined. The studies available have mostly focused on the link between empathy and adolescents’ attitudes toward out-group members. They have shown that adolescents with a lack of empathy are at risk of developing prejudiced beliefs toward out-group members (Miklikowska 2018). This is in agreement with earlier socio-cognitive theories, which indicate that individuals’ inability to understand another person’s perspectives may lead them to exaggerate the differences between themselves and members of an outgroup (Aboud 1988; Davis et al. 1996), and result in ethnic biases (Rutland and Killen 2015). By contrast, greater empathic capacity may help young people see that they and members of the other group have mutual similarities (Stephan and Finlay 1999). Empathic capacity can also help youth develop a good understanding of the possible challenges that their immigrant or minority peers may face. Thus, youth with greater empathic capacity may be more likely to stand-up against ethnicity-based injustices (Abbott and Cameron 2014), and less likely to engage in such behaviors themselves. However, there is no previous research that has empirically tested the link between empathy and ethnic victimization among adolescents. The present study aims to address this gap in knowledge.

#### The role of moral disengagement

Children become more cognitively mature during adolescence. Despite heterogeneity, acquiring cognitive skills generally helps adolescents to learn standards of moral conduct (e.g., Rutland and Killen 2015), and, in turn to be able to distinguish right from wrong in their social relationships, an ability conceptualized as *moral reasoning*. Bandura (1999) argued that moral reasoning is the cornerstone of (im)moral behaviors. However, he also emphasized that moral reasoning may not always lead to engagement in moral behaviors. That is, regardless of awareness of what is right and what is wrong, some people still engage in immoral behaviors, including acting aggressively or bullying others. Bandura et al. (1996) argued that self-regulatory cognitive processes may explain why or why not moral reasoning is translated into actual behavior. Specifically, they asserted that certain cognitive processes (i.e., cognitive restructuring leading to positive appraisals of bullying, minimizing one’s agentic role, disregarding or distorting the negative impact of harmful behavior, and blaming or dehumanizing the victim) may lead an individual to disengage from contextual morals, and behave unethically without shame, guilt, or truly experiencing the retribution of others. These cognitive processes are proposed as the mechanisms of moral disengagement.

Moral disengagement has been found to be an important indicator of antisocial behavior, including engagement in bullying across different age groups and in different social and cultural contexts (Pozzoli et al. 2012; Zych and Llorent 2019). Studies have indicated that children who bully or act aggressively toward others tend to perceive their victims as socially deviant (Teräsahjo and Salmivalli 2003; Thornberg and Knutsen 2011), and thereby believe that they deserve negative treatment. That is, the bullies tend to blame the victims so as to justify their own negative actions (Hymel et al. 2005; Thornberg and Jungert 2014). These children also have a greater tendency to cognitively restructure their harmful behaviors. For example, children who bully may think that it is acceptable to harm or hurt another person if their motive is to help or protect their own friends (Pozzoli et al. 2012; Thornberg and Jungert 2014). These justifications may lead children to disengage from individual moral control, to start viewing their negative actions as acceptable, and to make their behaviors habitual. In sum, the literature available suggests that children who victimize or bully their peers may, through cognitive processes, justify their immoral acts, and that such maladaptive cognitive processes may hinder them from seeing the possible negative consequences of their actions.

Being morally disengaged not only increases the risk that youth will bully or act aggressively, but may also determine how they view others of diverse backgrounds (D’errico and Paciello 2018; Passini 2019), and how they act in diverse settings (Mazzone et al. 2018). Previous studies of racism, particularly in relation to anti-immigrant attitudes and prejudiced beliefs, suggest that adults who are morally disengaged tend to hold derogatory and aversive attitudes toward out-group members (Passini 2019), and also to support racist acts (Faulkner and Bliuc 2016). Similar conclusions have been reached in a recent study focusing on adolescents. Specifically, a group of youth was presented with a hypothetical scenario in which a student was depicted as coming from another country, being excluded, and being teased by peers. The participants were then asked why the student might be treated in this manner. It was found that divergence from mainstream culture (e.g., inability to speak the local language, not being accustomed to cultural codes and values, or having a different religion) was reported as one of the reasons why the protagonist (i.e., an immigrant student) might have been victimized and excluded by peers (Mazzone et al. 2018). The findings suggest that many characteristics of immigrant youth may be perceived as “deviant”, and their maltreatment thereby justified. This further indicates that moral disengagement may be an important mechanism in the explanation of why some youth victimize their peers due to their ethnic or cultural background. However, no previous research has empirically tested the link between moral disengagement and ethnic victimization among adolescents. The present study aims to address this gap in knowledge.

#### The role of attitudes toward immigrants

Young people develop a set of beliefs about and attitudes to out-group members during their own social-identification process (Tajfel and Turner 1986). Some youth hold neutral or positive attitudes toward out-group members. By contrast, others negatively stereotype differences between their own in-group and out-groups, and perceive out-group members as unattractive, offensive, or inferior. This way of thinking may inflate these youth’s self-image and make them believe that they have a high social status simply by *not* belonging to an out-group. Negative stereotypes also play a crucial role in how youth interact with each other in school settings, and in the establishment of social harmony in schools (Dessel 2010). Relatedly, recent studies have shown that adolescents with negative views on out-group members (i.e., immigrants), or adolescents who perceive immigration as a social disadvantage for the host country, are more likely to engage in ethnic victimization (Bayram Özdemir et al. 2016; Bayram Özdemir et al. 2018) and racial bullying (Caravita et al. 2019). These findings suggest that youth’s prejudiced out-group perceptions may form the motivational grounds for bias-based aggressive acts.

Regarding the possible role of prejudiced beliefs and negative stereotypes in youth’s engagement in non-bias-based antisocial behaviors (including aggression or general victimization), the literature is rather limited and has provided mixed results. For example, in a study of high-school students in Italy, it was shown that, when adolescents had negative attitudes toward immigrants, they were more likely to endorse aggressive thoughts and behaviors in general (Piumatti and Mosso 2017). To the contrary, no relation between prejudiced beliefs and adolescents’ engagement in general bullying was reported in another study (Caravita et al. 2019). Further, Mazzone et al. (2018) found that, in Italian schools, adolescents reported that having an “unattractive” personality and/or deviating from mainstream appearance were possible reasons why both immigrant and native students had been victimized. Importantly, the same adolescents also reported that immigrant youth (*but not native youth*) might be victimized for other reasons, including ethnocultural difference and learned racism. In sum, the studies available suggest that holding prejudiced beliefs in conjunction with ethnic and cultural diversity may contribute to engagement in ethnic victimization to a greater extent than to general victimization.

## The Current Study

The current study aimed to further the understanding why youth engage in ethnic victimization and/or non-ethnicity-based victimization. The previous literature suggests that there is a moderate (*r* = 0.44; Caravita et al. 2019) to large (*r* = 0.54; Bayram Özdemir et al. 2016) association between ethnic victimization and non-ethnicity-based victimization, indicating that some youth might engage in both whereas others engage in either one of the two or none. However, it is unknown how these youth may otherwise differ. To address this gap in knowledge, the first goal of the present study was, by using a person-centered approach, to investigate whether there are distinct groups of adolescents who engage in ethnic and/or non-ethnicity-based victimization. The second goal was to elucidate the underlying factors that are common or unique to each group. Based on major conceptual arguments in the literature, we specifically examined the extent to which youth’s ability to regulate their behaviors (Gottfredson and Hirschi 1990), their ability to feel and understand others’ feelings (Davis 1983), the moral justification of their behaviors (Bandura 1999), and their attitudes toward immigrants (Bayram Özdemir et al. 2016) explain their engagement in ethnic and/or non-ethnicity-based victimization. It was expected that moral disengagement and lack of empathy would be common precursors of both ethnic and non-ethnicity-based victimization (Faulkner and Bliuc 2016; Mazzone et al. 2018; Rutland and Killen 2015). It was also expected that impulsivity would contribute to engagement in non-ethnicity-based victimization to a greater extent than in the perpetration of ethnic victimization (Bayram Özdemir et al. 2016; Näsi et al. 2016), and that the opposite would be the case for youth’s negative attitudes toward immigrants (Bayram Özdemir et al. 2018; Caravita et al. 2019).

## Methods

The sample for the current study comes from an ongoing 3-year longitudinal study—the Youth and Diversity Project. The main goal of the Youth and Diversity Project is to examine the role of school context in the development of positive and negative interactions among adolescents of diverse background. The project has been implemented in 55 classrooms in 16 different schools across four municipalities in central Sweden. In each school, 7th grade students (aged around 13) were targeted, and the target sample included 1286 adolescents. Of the target sample, 17% did not participate in the study for various reasons, including no consent from parents, no assent from students, and absence during data collection. A total of 1065 adolescents participated in the project. The analytical sample for the present study included only the adolescents with data on ethnic and non-ethnicity-based victimization (*N* = 949, *M*_age_ = 13.11, SD = 0.41; 46% girls).

About two-thirds of the adolescents (62%) had Swedish-born parents. The rest of the adolescents (38%) had at least one parent born outside Sweden, and were defined as youth with immigrant background. Among these youth, 37% were born outside Sweden (i.e., were first-generation immigrants). The length of stay in Sweden ranged from 1 to 12.5 years (*M* = 5.73; SD = 3.01) among these first-generation immigrant youth. About one-third of the immigrant adolescents (28%) reported speaking Swedish at home with their parents, while about one-third (22%) reported speaking another language, and more than half (50%) that they sometimes spoke Swedish and sometimes another language. The parents of the immigrant adolescents had migrated to Sweden from over 60 different countries, including Iraq, Iran, Somalia, Russia, Syria, Pakistan, Turkey, Bosnia, Kosovo, Germany, India, Italy, and the Netherlands. Among the immigrant youth, 39% reported that they attended a native language course inside or outside school. A majority of the adolescents (72%) came from an intact family and were living with both parents (72%). More than two-thirds of the adolescents reported that their parents were in employment (88% of mothers, and 94% of fathers).

### Procedure

A research manager and trained research assistants (who were university students) collected data from the 7^th^ grade students in the fall of 2018. The data collection took place in class and took about 90 min (i.e., two 45 min lessons). Before the data collection, parents were sent a letter with information about the study, and were asked to sign and return a form if they refused to allow their children to participate in the study. The information letter was written so that parents of different education levels could understand. Parents were also informed that they could request the information letter in other languages. Not returning the form in the information letter was interpreted as giving consent (i.e., passive consent). This procedure for obtaining consent is frequently used in developmental studies to increase participation and reduce sampling bias (Pokorny et al. 2001; Shaw et al. 2015). During the data-collection day, students were informed about the goals of the study, and were assured that their participation was voluntary, and that their responses would be confidential and not shared with anyone. Only the students whose parents did not decline their children’s participation and who themselves were willing to participate took part in the study. The questionnaire was administered in Swedish, but children with language difficulties received help from the research assistants in reading the questions. A sum of 500 Swedish crowns was given to each class in recognition of participation, and students were provided with snacks during data collection. The study procedures were approved by the Regional Research Ethics Committee in Uppsala (ref. number: Dnr 2018/235).

### Measures

#### Impulsivity

The Eysenck Impulsiveness Scale was used to measure adolescents’ level of impulsivity (Eysenck and Eysenck 1978). The scale consists of 4 items, and the sample items include statements like: “I usually do and say things without thinking about them” and “I mostly speak before thinking things through.” Adolescents were asked to report the extent to which they agreed or disagreed with the statements on a 5-point Likert scale ranging from “1” (strongly disagree) to “5” (strongly agree). The scale has been found to have high internal consistency and predictive validity (e.g., Dubas et al. 2017; Eysenck and Eysenck 1978). In the present study, Cronbach’s alpha for the scale was 0.74.

#### Empathy

A revised version of the Interpersonal Reactivity Index was used to measure adolescents’ empathic concern and perspective-taking skills in their peer relationships (Davis 1980). The empathic-concern subscale includes 4 items, which assess adolescents’ feelings of warmth, compassion, and concern for others. The sample items include the statements: “When I see someone being unfairly treated, it happens that I don’t feel particularly sorry for her/him (reverse item score)” and “It happens that I don’t feel sorry for other people when they have problems (reverse item score).” The perspective-taking subscale includes 5 items, which measure adolescents’ attempts to adopt the perspectives of other people and see things from their point of view. The sample items include the statements: “I try to understand others better by imagining how things look from their perspective” and “I believe that there are two sides to every question and try to look at them both.” Adolescents were asked to report how far each statement described them on a 5-point Likert scale, ranging from “1” (doesn’t describe me well at all) to “5” (describes me very well). This scale has been found to have good internal consistency and test-retest reliability (Davis 1980). In the present study, Cronbach’s alpha for the empathic-concern subscale was 0.56, and for the perspective-taking subscale 0.80.

#### Moral disengagement

A short version of the Moral Disengagement in Bullying Scale (Thornberg and Jungert 2014) was used to measure the degree to which adolescents morally disengage in bullying situations. The scale consists of 10 statements, and adolescents were asked to report how much they agreed or disagreed with each statement on a 5-point Likert scale, ranging from “1” (strongly disagree) to “5” (strongly agree). The sample items include: “It’s okay to hurt another person a couple of times a week if you do it to protect your friends” and “If people are weird, it’s their own fault that they get bullied.” The scale has been found to have high internal consistency and criterion validity (Thornberg and Jungert 2014). In the present study, Cronbach’s alpha for the scale was 0.91.

#### Positive attitudes toward immigrants

The Tolerance and Xenophobia Scale (van Zalk et al. 2013) was used to measure adolescents’ positive attitudes toward immigrants. The scale consists of 6 items, and the sample items include statements like: “Immigrants should have the same social rights as people born in Sweden” and “It is good for the Swedish economy that people move to Sweden”. Adolescents were asked to report on the extent to which they agreed or disagreed with these statements on a 5-point Likert scale, ranging from “1” (strongly disagree) to “5” (strongly agree). The scale has been found to have high internal consistency and predictive validity (e.g., van Zalk et al. 2013). In the present study, Cronbach’s alpha for the scale was 0.84.

#### Engagement in ethnic victimization

A four-item scale was used to measure youth’s engagement in ethnic victimization (i.e., being a perpetrator of ethnic victimization) (Bayram Özdemir and Özdemir 2020). The sample scale items were: “Have you said nasty things to anyone about her/his ethnic origin?” and “Have you excluded anyone from an activity because her/his parents came from another country? Adolescents were asked to respond to each question on a 5-point scale ranging from “1” (have not done that) to “5” (several times a week). The scale showed strong internal consistency in the present study (Cronbach’s alpha = 0.88).

#### Engagement in non-ethnicity-based victimization

The short version of the Linköping Bullying Scale (Thornberg and Jungert 2014) was used to measure adolescents’ engagement in non-ethnicity-based victimization. The scale consists of 6 items (e.g., “Teased or called the person nasty things” and “Spread nasty rumors or lies about the person”), and adolescents were asked to report how often they had engaged in these behaviors during the last 6 months. They answered on a 5-point scale (0 = “never,” 1 = “only occasionally,” 2 = “2 or 3 times a month,” 3 = “About once a week,” 4 = “Several times a week”). Previous research has provided evidence regarding the satisfactory internal consistency and concurrent validity of the scale (Thornberg and Jungert 2014). In the present study, Cronbach’s alpha for the scale was 0.79.

### Data Analysis

Hierarchical cluster analysis with squared Euclidian distance using the Ward method (Bergman 1998) was used to identify naturally occurring groups of youth who engage in ethnic and non-ethnicity-based victimization. Large increases in the agglomeration coefficients (using a scree plot), the explained error sum of squares for each cluster solution (minimum suggested value of 67%, Bergman 1998), and homogeneity within clusters and heterogeneity across clusters were the criteria employed to decide on the number of clusters in the final cluster solution (Hair et al. 2018). The robustness of the cluster solution was assessed by comparing hierarchical cluster solutions with non-hierarchical solutions (*K*-means clustering, Hair et al. 2018). Cluster analysis is highly sensitive to outliers and extreme values on the cluster indicators. Therefore, large *z*-transformed values of ethnic victimization (3.6%) and non-ethnicity-based victimization (2.3%) were recoded into the value of 2.5 (Bergman 1998). Next, the univariate differences in the study variables across the clusters were examined. This was followed up by performing a multinomial logistic regression to identify the unique predictors of the clusters, which referred to different constellations of youth based on their engagement in ethnic and non-ethnicity-based victimization of their peers. In all multinomial logistic regression models, adolescents’ age, gender, and immigrant background were controlled for. The expectation-maximization (EM) method was used to handle missing data. This method provides unbiased estimates compared with traditional techniques (e.g., listwise deletion, pairwise deletion, or mean imputation). It eliminates Type-II errors, and does not underestimate correlations and standardized regression coefficients (Acock 2005; Kline 2011).

## Results

### Hierarchical Cluster Analysis

**Fig. 1:**
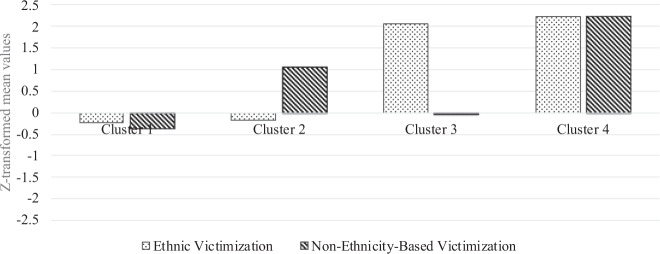
Bar-chart of *z*-transformed mean values of ethnic and non-ethnicity-based victimization by cluster

Means, standard deviations and correlations among the study variables are presented in Table 1. Hierarchical cluster analysis was used to identify whether there are distinct groups of adolescents who engage in ethnic and/or non-ethnicity-based victimization. The point-of-inflection method indicated a four-cluster solution (Hair et al. 2018). The four clusters explained 78% of the error sum of squares, which is higher than the suggested level of 67% for identifying homogeneous clusters (Bergman 1998). Due to violation of the homogeneity of variance assumption, Welch *F*-tests were performed to examine cluster heterogeneity. The clusters were sufficiently differentiated on both ethnic victimization, *F*(3, 66.53) = 516.46, *p* < 0.001, *η*^2^ = 0.82, and non-ethnicity-based victimization, *F*(3, 67.12) = 806.70, *p* < 0.001, *η*^2^ = 0.77 (see Table 2 and Fig. 1). The cluster solution from the hierarchical cluster analysis was compared with the *k*-mean clustering solution (Hair et al. 2018). The comparison suggested that the cluster solution was robust, as indicated by a high Kappa value of 0.94, and 98% agreement between the two cluster solutions.

**Table 1:** Bivariate Correlations, Means, and Standard Deviations of the Study Variables

	1	2	3	4	5	6	7	8	9	10
1. Ethnic victimization	–									
2. Non-ethnic victimization	0.59^***^	1								
3. Age	0.14^***^	0.11^***^	–							
4. Gender^1^	0.22^***^	0.25^***^	0.08^**^	–						
5. Immigrant background^2^	0.11^***^	0.12^***^	0.12^***^	0.02	–					
6. Empathic concern	−0.29^***^	−0.25^***^	−0.08^**^	−0.27^***^	0.02	–				
7. Perspective taking	−0.02	−0.11^***^	−0.03	−0.14^***^	0.03	0.22^***^	–			
8. Impulsivity	0.15^***^	0.32^***^	−0.02	0.10^**^	0.03	−0.22^***^	−0.25^***^	–		
9. Attitudes toward immigrants	−0.15^***^	−0.11^***^	−0.08^**^	−0.10^***^	0.09^**^	0.27^***^	0.36^***^	−0.12^***^	–	
10. Moral disengagement	0.43^***^	0.45^***^	0.16^***^	0.29^***^	0.09^**^	−0.40^***^	−0.18^***^	0.36^***^	−0.19^***^	–
*M*	1.15	1.28	13.11	0.54	0.38	3.61	3.21	2.68	3.57	1.69
SD	0.47	0.43	0.40	0.50	0.49	0.67	0.73	0.77	0.78	0.65
Min	1	1	12	0	0	1	1	1	1	1
Max	5	5	15	1	1	5	5	5	5	5

*p < 0.05; **p < 0.01; ***p < 0.001

^1^Gender was coded as 0 = Girls and 1 = Boys

^2^Immigrant background was coded as 0 = Swedish and 1 = Immigrant

**Table 2:** Means, Standard Deviations, 95th Percentile Confidence Intervals for Ethnic and Non-ethnicity-based Victimization by Cluster

			Ethnic Victimization			Non-ethnicity-based Victimization		
	*N*	%	*M*	SD	95% CI	*M*	SD	95% CI
Cluster 1—LL	746	78.6	−0.23^a^	0.25	−0.25, −0.21	−0.37^a^	0.01	−0.39, −0.34
Cluster 2—LH	143	15.1	−0.18^a^	0.29	−0.22, −0.13	1.05^b^	0.55	0.96, 1.14
Cluster 3—HL	25	2.6	2.07^b^	0.46	1.87, 2.25	−0.05^c^	0.59	−0.29, 0.19
Cluster 4—HH	35	3.7	2.24^c^	0.46	2.08, 2.40	2.24^d^	0.37	2.11, 2.5
Welch *F-*test			516.46			806.70		
* df*			3, 66.53			67.12		
* p*			<0.001			<0.001		
* η*^2^			0.82			0.77		

*LL* = low ethnic and non-ethnicity-based victimization, *LH* = low ethnic, high non-ethnicity-based victimization, *HL* = high ethnic, low non-ethnicity-based victimization, *HH* = high ethnic and non-ethnicity-based victimization

^a, b, c, d^Superscripts denotes the statistically significant group differences, based on Student–Newman–Keuls (S–N–K) post-hoc comparisons of group means

78.6% of the sample showed low levels of engagement in both ethnic and non-ethnicity-based victimization. 15.1% of the adolescents reported high levels of engagement in non-ethnicity-based victimization and low levels of engagement in ethnic victimization. About 2.6% of the adolescents reported high levels of engagement in ethnic victimization and low levels of engagement in general victimization. Finally, 3.7% of the adolescents reported high levels of engagement in both ethnic and non-ethnicity-based victimization. The univariate differences across the clusters on the study variables, including the demographic characteristics, are shown in Table 3.

**Table 3:** Differences across the Clusters on the Study Variables

	Cluster 1—LL		Cluster 2—LH		Cluster 3—HL		Cluster 4—HH					
	*M*	SD	*M*	SD	*M*	SD	*M*	SD	Welch *F*	*df*	*p*	*η*^2^
Age	13.10^a^	0.39	13.15^a, b^	0.44	13.27^a, b^	0.43	13.34^b^	0.47	4.608	3, 68.45	0.005	0.02
Gender^1^	0.48^a^	0.5	0.72^b^	0.46	0.92^c^	0.29	0.95^c^	0.24	51.261	3, 74.31	<0.001	0.07
Immigrant background^2^	0.36^a^	0.48	0.46^a^	0.5	0.35^a^	0.49	0.59^a^	0.5	3.617	3, 64.95	0.018	0.02
Empathic concern	3.70^a^	0.66	3.42^b^	0.57	3.01^c^	0.65	3.05^c^	0.62	26.567	3, 70.09	<0.001	0.08
Perspective-taking skills	3.29^a^	0.72	2.92^b^	0.61	2.91^b^	0.75	3.11^a, b^	0.93	14.912	3, 69.01	<0.001	0.04
Impulsivity	2.58^a^	0.76	3.12^b^	0.73	2.66^a^	0.71	3.23^b^	0.57	31.757	3, 71.22	<0.001	0.08
Attitudes toward immigrants	3.65^a^	0.75	3.44^a, b^	0.77	2.73^c^	0.95	3.23^b^	0.89	11.826	3, 68.25	<0.001	0.05
Moral disengagement	1.56^a^	0.54	2.02^b^	0.67	2.34^c^	0.72	2.71^d^	1.07	39.203	3, 66.91	<0.001	0.19

^1^Gender was coded as 0 = Girls, 1 = Boys

^2^Immigrant background was coded as 0 = Swedish, 1 = Immigrant

*LL* = low ethnic and non-ethnicity-based victimization, *LH =* low ethnic, high non-ethnicity-based victimization, *HL=* high ethnic, low non-ethnicity-based victimization, *HH* = high ethnic and non-ethnicity-based victimization

^a, b, c, d^Superscripts denotes the statistically significant group differences based on Student–Newman–Keuls (S–N–K) post-hoc comparisons of group means

### Predictors of Cluster Membership

Two multinomial logistic regression models were fitted to examine the extent to which youth’s ability to regulate their behaviors, their ability to feel and understand others’ feelings, the moral justification of their behaviors, and their attitudes toward immigrants predicted cluster membership. In the first model, adolescents who had low levels of engagement in both non-ethnicity-based and ethnic victimization were defined as the reference group. This model allowed us to see what predicts being in one of the non-normative clusters (i.e., high non-ethnicity-based victimization, high ethnic victimization, and high on both forms of victimization) relative to being in the normative cluster (i.e., the low-victimization group) (see Table 4). In the second model, adolescents who engaged in high ethnic victimization only were defined as the reference cluster so as to compare their cluster with the high non-ethnicity-based victimization and high ethnic and non-ethnicity-based victimization clusters (see Table 5).

**Table 4:** Multinomial Logistic Regression Results Comparing Three Victimization Clusters with the Low Ethnic and Non-ethnicity-based Victimization Cluster

	LH vs. LL			HL vs. LL			HH vs. LL		
	*B* (SE)	*p*	*Exp* (B)	*B* (SE)	*p*	*Exp* (B)	*B* (SE)	*p*	Exp (B)
Age	0.17 (0.25)	0.497	1.18	0.29 (0.50)	0.566	1.33	0.57 (0.42)	0.177	1.75
Gender^1^	0.71 (0.22)	<0.001	2.02	1.69 (0.78)	0.029	5.41	2.23 (0.77)	0.003	9.29
Immigrant background^2^	0.45 (0.21)	0.026	1.57	0.24 (0.49)	0.624	1.27	1.06 (0.41)	0.010	2.88
Empathic concern	−0.17 (0.18)	0.343	0.85	−0.82 (0.39)	0.034	0.44	−0.49 (0.36)	0.170	0.61
Perspective-taking skills	−0.45 (0.16)	0.004	0.64	−0.22 (0.34)	0.510	0.80	−0.07 (0.29)	0.833	0.94
Impulsivity	0.71 (0.15)	<0.001	2.02	−0.42 (0.36)	0.248	0.66	0.69 (0.29)	0.017	1.98
Attitudes toward immigrants	−0.07 (0.14)	0.650	0.94	−0.96 (0.27)	<0.001	0.39	−0.62 (0.25)	0.013	0.54
Moral disengagement	0.65 (0.18)	<0.001	1.91	1.22 (0.34)	<0.001	3.38	1.28 (0.28)	<0.001	3.57

^1^Gender was coded as 0 = Girls, 1 = Boys

^2^Immigrant background was coded as 0 = Swedish, 1 = Immigrant

*LL=* low ethnic and non-ethnicity-based victimization, *LH* = low ethnic, high non-ethnicity-based victimization, *HL* = high ethnic, low non-ethnicity-based victimization, *HH* = high ethnic and non-ethnicity-based victimization

Reference group: Low ethnic and non-ethnicity-based victimization

Model fit information: Likelihood ratio test, *χ*^2^ (24) = 271.07, *p* < 0.001; Hosmer–Lemeshow test, *χ*^2^ (2802) = 2508.27, *p* = 1.000; Nagelkerke *R*^2^ = 0.33

**Table 5:** Multinomial Logistic Regression Results Comparing Three Clusters with the High Ethnic Victimization Cluster

	LL vs. HL			LH vs. HL			HH vs. HL		
	*B* (SE)	*p*	*Exp* (B)	*B* (SE)	*p*	*Exp* (B)	*B* (SE)	*p*	*Exp* (B)
Age	−0.29 (0.50)	0.566	0.75	−0.12 (0.52)	0.819	0.89	0.28 (0.60)	0.638	1.32
Gender^1^	−1.69 (0.78)	0.029	0.19	−0.99 (0.80)	0.213	0.37	0.54 (1.08)	0.614	1.72
Immigrant background^2^	−0.24 (0.49)	0.624	0.79	0.22 (0.50)	0.670	1.24	0.83 (0.59)	0.162	2.27
Empathic concern	0.82 (0.39)	0.034	2.26	0.65 (0.41)	0.107	1.92	0.33 (0.49)	0.502	1.39
Perspective-taking	0.22 (0.34)	0.510	1.25	−0.23 (0.35)	0.52	0.80	0.16 (0.41)	0.695	1.17
Impulsivity	0.42 (0.36)	0.248	1.52	1.12 (0.38)	0.003	3.06	1.10 (0.44)	0.011	3.01
Attitudes toward immigrants	0.96 (0.27)	<0.001	2.59	0.89 (0.29)	0.002	2.43	0.34 (0.34)	0.311	1.40
Moral disengagement	−1.22 (0.34)	<0.001	0.30	−0.57 (0.35)	0.098	0.57	0.06 (0.39)	0.885	1.06

^1^Gender was coded as 0 = Girls, 1 = Boys

^2^Immigrant background was coded as 0 = Swedish, 1 = Immigrant

*LL* = low ethnic and non-ethnicity-based victimization, *LH* = low ethnic, high non-ethnicity-based victimization, *HL* = high ethnic, low non-ethnicity-based victimization, *HH* = high ethnic and non-ethnicity-based victimization

Reference group: High ethnic victimization cluster

Model fit information: Likelihood ratio test, *χ*^2^ (24) = 271.07, *p* < 0.001; Hosmer–Lemeshow test, *χ*^2^ (2802) = 2508.27, *p* = 1.000; Nagelkerke *R*^2^ = 0.33

#### Comparison between the normative cluster and the non-normative clusters

The results from the first multinomial logistic regression model showed that adolescents in the high non-ethnicity-based victimization cluster had a greater likelihood of being male and having an immigrant background than those in the normative cluster. In addition, these adolescents had a lower likelihood of having perspective-taking skills, and a greater likelihood of displaying impulsivity and being morally disengaged. Adolescents’ age, empathic concerns, and attitudes toward immigrants did not significantly differentiate the adolescents in the high general-victimization cluster from those in the normative cluster (see Table 4).

In the comparison between the high ethnic victimization cluster and the normative cluster, it was found that adolescents in the high ethnic victimization cluster had a greater likelihood of being male and being morally disengaged. Importantly, these adolescents also had a lower likelihood of having empathic concerns and positive attitudes toward immigrants. Adolescents’ age, immigrant background, perspective-taking skills, and impulsivity did not significantly differentiate the adolescents in the high ethnic victimization cluster from those in the normative cluster (see Table 4).

Finally, the adolescents in the high ethnic and non-ethnicity-based victimization clusters were compared with those in the normative cluster. The results showed that adolescents in the high ethnic and non-ethnicity-based victimization cluster had a greater likelihood of being male and immigrant. They also had a greater likelihood of being impulsive and morally disengaged, and a lower likelihood of having positive attitudes toward immigrants. Adolescents’ age, empathic concerns, and perspective-taking skills did not significantly differentiate the adolescents in this cluster from those in the normative cluster (see Table 4).

In sum, the results from the first multinomial logistic regression model indicated that the predictors of being in non-normative clusters relative to being in the normative cluster have both similarities and dissimilarities. Being male and morally disengaged seems to be a common denominator of all three non-normative clusters. Having an immigrant background and displaying impulsivity were the common risk factors for both clusters high on non-ethnicity-based victimization. Low positive attitudes toward immigrants was the risk factor shared by both the clusters high on ethnic victimization.

#### Comparisons between the non-normative clusters

As previously stated, in the second multinomial logistic regression model, the high ethnic victimization cluster was defined as the reference cluster, which was then compared with the other two non-normative clusters (i.e., high on non-ethnicity-based victimization and high on both forms of victimization) (Table 5). The results showed that adolescents in the high non-ethnicity-based victimization cluster had a greater likelihood of being impulsive and having positive attitudes toward immigrants than those in the high ethnic victimization cluster. None of the other predictors significantly differentiated these two clusters (see Table 5). Then, the adolescents in the high ethnic and general victimization cluster were compared with those in the high ethnic victimization cluster. The results showed that adolescents in the high ethnic and general victimization cluster had a greater likelihood of being impulsive. None of the other predictors significantly differentiated these two clusters (see Table 5). In sum, the findings suggest that impulsivity is a common denominator of both the clusters that are high on non-ethnicity-based victimization, but is not a risk factor for engaging in ethnic victimization exclusively. Adolescents who engaged solely in ethnic victimization had lower levels of positive attitudes toward immigrants than those who engaged in non-ethnicity-based victimization exclusively.

## Discussion

Societies are becoming more ethnically and culturally diverse. This increasing diversity brings new opportunities to both individuals and immigrant-receiving societies. But it also poses challenges, such as a polarized political climate and an increase in bias-based hostile behaviors, such as ethnic discrimination and victimization. As shown across multiple studies, ethnicity-based negative treatments can have detrimental consequences for immigrant and minority adolescents’ psychosocial functioning and behavioral adjustment (e.g., Bayram Özdemir and Stattin 2014; McKenney et al. 2006). Despite a growing body of research into this societal concern, the majority of existing research has adopted a victim perspective that aims to understand the consequences of being a target of negative treatment. By contrast, relatively little attention has been paid to understanding who the perpetrators of ethnic victimization are (e.g., Bayram Özdemir et al. 2019; Caravita et al. 2019). This gap in knowledge prompts the question of whether the youth who victimize their peers due to their ethnic background are also the ones who engage in non-ethnicity-based victimization. Using a person-centered approach, this study investigated whether there are distinct groups of adolescents who engage in ethnic and/or non-ethnicity-based victimization. Further, it elucidated the underlying factors that are common or unique to each group, by focusing on the roles of impulsivity, empathetic concern and perspective-taking, moral disengagement, and attitudes toward immigrants.

Four distinct groups of youth were identified, based on their levels of engagement in ethnic and/or non-ethnicity-based victimization. More than two-thirds of the adolescents (79%) were in the normative group, meaning that they reported no engagement in any form of victimization. About 15% of the adolescents reported engaging in *only* non-ethnicity-based victimization, and 3% of them reported engaging in *only* ethnic victimization. About 4% of the adolescents reported engaging in both forms of victimization. Thus, some youth seem to be at risk of engaging in both forms of victimization, whereas others may only engage in either ethnic or non-ethnicity-based victimization. The findings also indicate that there are relatively more adolescents involved in non-ethnically based victimization than in ethnicity-based victimization, which might be related to the motives of different types of peer victimizers. Specifically, ethnicity-based victimization has a clear motive, founded in prejudiced beliefs (Bayram Özdemir et al. 2016; Caravita et al. 2019) or low tolerance toward immigrants (Bayram Özdemir and Özdemir 2020). In the current data, only 5% of adolescents had low positive attitudes toward immigrants (i.e., the percentage of adolescents who scored 2 or below on the positive attitudes toward immigrant scale). The small proportion of adolescents with low positive attitudes toward immigrants might be one of the reasons why there are relatively few adolescents in the ethnicity-based victimization cluster (2.6% of the sample). By contrast, non-ethnicity-based victimization might occur as a function of different motives, making this form of peer victimization more prevalent than non-ethnicity-based victimization. Supporting this argument, in a large-scale study in Finland, it was found that 15% of adolescents reported engaging in global bullying, whereas only 4% of them reported engaging in racist bullying (Salmivalli et al. 2011). Taken as a whole, it can be argued that differences in the motives underlying ethnicity-based and non-ethnicity-based victimization might account, at least in part, for why there are relatively more adolescents involved in non-ethnically-based victimization than ethnicity-based victimization.

A noteworthy contribution of this study lies in its simultaneous examination of multiple factors that might predispose youth to engage in different forms of victimization. The findings show that there are certain common precursors to ethnic and non-ethnicity-based victimization. Specifically, it was found that adolescents in the non-normative groups (i.e., high on ethnic victimization, high on non-ethnicity-based victimization, and high on both forms of victimization) tend to show greater moral disengagement than those in the normative group. No significant differences in moral disengagement were observed across any of the non-normative groups. This finding indicates that moral disengagement not only puts young people at risk of engagement in non-ethnicity-based victimization (e.g., Gini et al. 2015; Patrick et al. 2019), but also elevates their risk of engagement in ethnic victimization (Faulkner and Bliuc 2016). It is likely that, regardless of the forms of victimization in which youth engage, many see their victims as socially deviant, and believe that the victims deserve the treatment they get. Such cognitive processes might decouple these youth from contextual morals, and make them believe that their negative actions are acceptable. Thus, they may become incapable of thoroughly understanding the consequences for their peers of their own misbehaviors. Together, these findings highlight the importance of targeting immoral cognitive justifications in attempts to prevent youth engagement in non-ethnicity-based victimization and to reduce inter-ethnic conflicts between young people in culturally diverse schools.

Importantly, the findings also suggest that there are unique, separate factors that explain why youth engage in ethnic and non-ethnicity-based victimization. For example, it was found that adolescents who engage in non-ethnicity-based victimization are more likely to be impulsive than those who engage in ethnic victimization only. Supporting previous research (Näsi et al. 2016), this finding suggests that while impulsivity may be an important explanatory mechanism in engagement in general victimization, it seems to play a lesser role in engagement in ethnic victimization. It is possible that non-ethnicity-based victimization occurs “in the heat of the moment” whereas ethnic victimization is a more deliberate behavior. Thus, poor self-control may not be a critical precursor of ethnic victimization in itself, especially when other risk factors are considered.

Another important finding of the study is that adolescents who engage in ethnic victimization, or who engage in both forms of victimization, are less likely to hold positive attitudes toward immigrants than those who engage in non-ethnicity-based victimization only. This finding holds even after controlling for other risk factors. Supporting previous research (Bayram Özdemir et al. 2018; Caravita et al. 2019), it suggests that views on immigrants may form the motivational base for engagement in ethnic victimization among adolescents, and further highlights the importance of fostering positive attitudes toward immigrants as a target for programs aiming to reduce ethnic victimization in schools.

The findings regarding the role of empathy in youth’s engagement in ethnic or non-ethnicity-based victimization show a complex pattern of associations. Supporting previous research (e.g., Dinić et al. 2016; van Noorden et al. 2017), the univariate analysis shows that adolescents in any of the non-normative groups (i.e., high ethnic victimization, high non-ethnicity-based victimization, or high on both forms of victimization) have lower empathic concerns and perspective-taking skills than those in the normative group. However, this clear pattern of group differences disappears when the roles of other potential risk factors (i.e., impulsivity, moral disengagement, and attitudes toward immigrants) are taken into account. Specifically, the results show that adolescents who engage solely in non-ethnicity-based victimization have lower levels of perspective-taking skills than normative youth. This finding is in line with the previous literature (e.g., Dinić et al. 2016), and suggests that youth who victimize or act aggressively toward their peers are incapable of recognizing the emotional states of their peers and adopting their perspectives. It is possible that difficulties in recognizing the viewpoints of another person might put these youth at risk of making erroneous judgments about their peers’ behaviors and intentions, and might therefore increase the likelihood of making hostile attributions, and, in turn, engaging in victimization.

Importantly, it was also found that, relative to normative adolescents, those who only engage in ethnic victimization have lower levels of empathic concern, and that there is no difference between the two groups regarding perspective-taking skills. This finding suggests that the perpetrators of ethnic victimization may not necessarily have difficulties in understanding the perspectives of their peers, but rather lack the emotional ability to participate in others’ emotions. Thus, they may be incapable of experiencing their victims’ pain and have inadequate insight into how victimization experiences may impact their immigrant or minority peers (Abbott and Cameron 2014).

Interestingly and unexpectedly, the findings also revealed that empathic concerns and perspective-taking skills do not significantly differentiate adolescents who engage in both forms of victimization from those in the normative group. One possible explanation for this is that adolescents who engage in multiple forms of victimization have a well-developed theoretical understanding of people’s minds and are able to understand their peers’ mental states. However, they may use their social intelligence manipulatively, and thus are more at risk of engagement in immoral conduct (Sutton et al. 1999). Altogether, these findings further indicate that the relation between empathy and engagement in different forms of victimization is not clear-cut, especially when other risk factors are also considered.

Another noteworthy finding of the study is that, while being male is a common denominator of all three non-normative groups, having an immigrant background is only a risk factor for engagement in non-ethnicity-based victimization, and engagement in both forms of victimization. The observed gender difference is consistent with previous research showing that boys are more at risk of engagement in both general (e.g., Sentse et al. 2015) and ethnic victimization (e.g., Bayram Özdemir and Özdemir 2020). One possible explanation for the gender difference is related to the differences in emotional and cognitive skills between males and females. Female adolescents have been consistently found to have more advanced emotional and cognitive skills than males (for a meta-analysis, see Chaplin and Aldao 2013; Tucker Smith et al. 2016), may be better aware of the possible consequences of victimization for its targets, and are thereby less inclined to treat their peers in this way. The finding regarding the role of immigrant background is also in line with previous studies showing that youth of immigrant background are at greater risk of victimizing their peers (Fandrem et al. 2009). This finding may be related to the fact that being immigrant is likely to be accompanied by other risk factors, such as low socio-economic status (SES), a greater likelihood of attending a school with a chaotic climate, and a greater likelihood of being victimized. These unmeasured factors might elevate the risk of engagement in victimization among immigrant youth. In sum, future research is needed to empirically test these conceptual arguments in order better to explain why males and adolescents of immigrant background are more at risk of engaging in different forms of victimization.

Despite its important contributions to the literature, several limitations of the present study need to be acknowledged. First, given that understanding youth’s own perceptions was the main interest of the present study, adolescents’ self-reports were used in the assessment of all study constructs. This approach might raise some concerns. For example, it is unknown how adolescents who define themselves as empathic are perceived by their peers. There may be a risk that one’s own appraisals of empathy are not representative of others’ experiences of one’s empathetic responses. Corroborating adolescents’ self-reports with data from peers would contribute to the literature in this regard. Second, the study presented here was correlational by nature, and the data captured only one time point. This prevents the investigation of whether youth who are identified in the normative group move to one of the non-normative groups over time or vice-versa, and the factors that explain these possible changes. Future studies with multi-year longitudinal data are needed to address this limitation. Third, the internal consistency value of the emphatic concern subscale was below the recommended criterion. This constraint might result in the making of a Type-II error. Thus, the findings about empathic concern should be interpreted cautiously, and need to be replicated using other empathy measures that have been shown to be reliable and valid among adolescents (Jolliffe and Farrington 2011). Fourth, the focus of the current study was on identifying individual-level risk factors that might play a role in youth’s engagement in ethnic and/or non-ethnicity-based victimization. However, recent studies have shown that not only individual factors but also contextual factors (e.g., school climate and the mass media) may contribute to youth’s engagement in different forms of victimization (e.g., Bayram Özdemir and Özdemir 2020). Examining the extent to which school and family contexts explain youth engagement in ethnic and/or non-ethnicity-based victimization would advance knowledge in the field. Finally, the present study focused on overt peer victimization. However, recent studies have suggested that immigrant and minority youth are not only exposed to overt ethnicity-based victimization (e.g., racist name-calling), but also face subtle forms of peer victimization, such as microaggression (Huynh 2012). Identifying the precursors of both overt ethnicity-based victimization and microaggression, and examining whether *and* when microaggression turns into overt peer victimization, would be informative in preventing negative interactions among adolescents of diverse backgrounds in school.

## Conclusions

Adolescents may engage in different forms of victimization of their peers. Despite the overlap between ethnicity and non-ethnicity-based victimization, prior studies have provided evidence suggesting that not all adolescents engage in both of these non-normative behaviors. Nevertheless, there has been a lack of understanding of the characteristics of the youth who engage in the separate or both forms of victimization. The present study sheds light on the question of whether youth who victimize their peers due to their ethnic background are also the ones who engage in non-ethnicity-based victimization, and provides empirical evidence on the underlying factors that are common or unique to each group. The findings suggest that some youth are at risk of engaging in both forms of victimization, whereas others only engage in either ethnic or non-ethnicity-based victimization. It also shows that the precursors of engagement in ethnic and non-ethnicity-based victimization show similarities as well as differences. Being male and morally disengaged seems to be a common denominator of ethnic and non-ethnicity-based victimizers. While a lack of ability to regulate behavior seems to trigger youth’s engagement in non-ethnicity-based victimization, attitudes toward immigrants provide the motivational base for ethnic victimization. Together, the methodological and conceptual approaches taken in this study elucidate the potential benefit of examining common and distinct predictors of different types of peer victimization simultaneously. The findings contribute to the advancement of understanding of which adolescents engage in both particular and multiple forms of victimization and why. The results indicate that programs aimed at preventing ethnicity or non-ethnicity-based peer victimization may do well to target moral disengagement, and that attitudes and values rather than self-regulatory skills may be of importance when targeting ethnic victimization.
